# Deprived TLR9 Expression in Apparently Healthy Nasal Mucosa Might Trigger Polyp-Growth in Chronic Rhinosinusitis Patients

**DOI:** 10.1371/journal.pone.0105618

**Published:** 2014-08-18

**Authors:** Lotta Tengroth, Julia Arebro, Susanna Kumlien Georén, Ola Winqvist, Lars-Olaf Cardell

**Affiliations:** 1 Division of Ear, Nose and Throat Diseases, Department of Clinical Sciences, Intervention and Technology, Karolinska Institutet, Stockholm, Sweden; 2 Department of Medicine, Unit of Translational Immunology, Karolinska Institute, Stockholm, Sweden; Centre d'Immunologie de Marseille-Luminy, CNRS-Inserm, France

## Abstract

**Background:**

The origin of nasal polyps in chronic rhinosinusitis is unknown, but the role of viral infections in polyp growth is clinically well established. Toll-like receptors (TLRs) have recently emerged as key players in our local airway defense against microbes. Among these, TLR9 has gained special interest in viral diseases. Many studies on chronic rhinosinusitis with nasal polyps (CRSwNP) compare polyp tissue with nasal mucosa from polyp-free individuals. Knowledge about changes in the turbinate tissue bordering the polyp tissue is limited.

**Objectives:**

To analyse the role of TLR9 mediated microbial defense in tissue bordering the polyp.

**Methods:**

Nasal polyps and turbinate tissue from 11 patients with CRSwNP and turbinate tissue from 11 healthy controls in total were used. Five biopsies from either group were analysed immediately with flow cytometry regarding receptor expression and 6 biopsies were used for *in vitro* stimulation with a TLR9 agonist, CpG. Cytokine release was analysed using Luminex. Eight patients with CRSwNP in total were intranasally challenged with CpG/placebo 24 hours before surgery and the biopsies were collected and analysed as above.

**Results:**

TLR9 expression was detected on turbinate epithelial cells from healthy controls and polyp epithelial cells from patients, whereas TLR9 was absent in turbinate epithelial cells from patients. CpG stimulation increased the percentage cells expressing TLR9 and decreased percentage cells expressing VEGFR2 in turbinate tissue from patients. After CpG stimulation the elevated levels of IL-6, G-CSF and MIP-1β in the turbinate tissue from patients were reduced towards the levels demonstrated in healthy controls.

**Conclusion:**

Defects in the TLR9 mediated microbial defense in the mucosa adjacent to the anatomic origin of the polyp might explain virus induced polyp growth. CpG stimulation decreased VEGFR2, suggesting a role for CpG in polyp formation. The focus on turbinate tissue in patients with CRSwNP opens new perspectives in CRSwNP-research.

## Introduction

Chronic rhinosinusitis (CRS) constitutes a public health problem with a negative impact on quality of life [1]. According to recent nomenclature, CRS can be subdivided into CRS with or without nasal polyps (CRSwNP or CRSsNP) [1]. The pathogenesis of CRS, as well as the origin of polyp development, still remains substantially unknown, even though numerous inflammatory signs and markers that have been associated with the disease [2]. Several studies have shown abnormalities in the immune responses in patients with CRSwNP [3], [4]. Defects in the host response to external pathogens including virus, bacteria and fungi have been suggested to underlie the persistence of the inflammatory state [5]. Clinically, respiratory viral infections are often implicated as triggers of CRS flare-ups and these infections are also known to damage the function of nasal epithelial cells and cilia [6], [7]. Viral infections of the airway are also shown to be followed by bacterial infections. For example, Herpes simplex virus type 1 expressed in nasal polyps results in damaging effects to the epithelium, facilitating invasion of *Staphylococcus aureus* [8]. *Staphylococcus aureus* has further been shown to play a role in CRSwNP by releasing enterotoxins that acts as superantigens, amplifying a T helper 2 (Th2)-biased immune response [9].

Pattern-recognition receptors (PRRs) on the nasal mucosa have the ability to detect and bind conserved microbial components and initiate an immune response. The most well-known members of the PRR family are the Toll-like receptors (TLRs) [10]. Mammals express at least 10 TLRs that recognize specific pathogen molecules [3]. TLR9, a receptor of the TLR family, induces a T helper 1 (Th1)-biased immune response that is known to suppress Th2-related activities, associated with CRSwNP [11]. Cytosine-phosphate-guanine oligodeoxynucleotide (CpG-ODN) is a TLR9 ligand found in bacterial and viral DNA. The ability for CpG to induce Th1-polarization has made it an interesting novel target for the treatment of allergy and infectious diseases [12], [13].

We have previously demonstrated TLR9 expression in nasal mucosa from healthy individuals [14]. It appears to be most prominently expressed in the epithelium, but it can also be found on leukocytes scattered in the intraepithelial and subepithelial layer [14]. A decreased expression of TLR9 mRNA has been found in sinus mucosa from patients with recurrent CRSwNP, compared to patients with milder disease [15] and this might be linked to the increased effects of locally produced Th2 cytokines in CRSwNP [15], [16]. However, many studies in the polyp field have focused on comparing polyp tissue with turbinate tissue from healthy, polyp-free, individuals. Knowledge about changes in the apparently healthy turbinate tissue close to the anatomic origin of the polyp is scarce. It is limited to only a few reports regarding inflammatory cell counts and cellular proliferation activities [17], [18]. The aim of the present study is to uncover novel approaches for CRSwNP research by focusing on the role of TLR9 in apparently healthy turbinate tissue bordering the polyp tissue.

## Materials and Methods

### Ethics Statement

The study was approved by the Ethics Committees of Karolinska Institutet, Stockholm, Sweden (2012/1482–31/1). All participants, those biopsied with or without intranasal challenge with CpG, gave their written informed consent, while all procedures were conducted according to the principles expressed in the Declaration of Helsinki.

### Subjects and Study design

The patients with CRSwNP were defined by historical and endoscopic criteria and/or CT changes (accordingly to the European Position Paper on Rhinosinusitis and Nasal Polyps guidelines [1]). In all patients, steroids were withheld during at least 6 weeks (topically) and 12 weeks (systemically) prior to functional endoscopic sinus surgery (FESS). Patients on daily inhaled steroid medication, as well as those with more than four episodes of FESS, were excluded from the study. None of the patients had history of smoking, 6 out of 19 had history of asthma and 9 out of 19 had a positive phadiatop test, with presence of serum-specific IgE towards one or several allergens in the standard panel (birch, mugwort, timothy, dog, cat, horse, mites and mold).

Biopsies from patients with CRSwNP were taken from the polyp tissue and the apparently healthy, turbinate tissue, during FESS. The area close to middle nasal meatus, from the middle turbinate or the inferior turbinate, where the mucosa had healthy appearance and bordered to the polyp tissue or to the tissue showing polypoidal changes, was defined as the turbinate tissue. The healthy controls had no history of polyp disease or smoking history and had negative phadiatop test. Biopsies were obtained as previously described [19].

Biopsies from 11 patients with CRSwNP (9 males and 2 females with a mean age of 51, range 18–84), and 11 healthy controls (5 males and 6 female with a mean age of 28, range 20–48) were used in total, 5 biopsies were analysed immediately with flow cytometry and 6 biopsies used for *in vitro* stimulation experiments. Further, 8 additional patients with CRSwNP were included in the study with intranasal challenge of CpG. These patients were randomized to receive either CpG (*n* = 4; 2 males and 2 females with a mean age of 44, range 32–56) or placebo (*n* = 4; 3 males and 1 female with a mean age of 62, range 56–71).

### *In vitro* stimulation with CpG

Biopsies used for *in vitro* stimulation were collected into sterile DMEM/F-12 (1X) (Gibco, Paisley, UK) and separated into equally small pieces of 0.05 g. The tissue pieces were incubated on 24-well culture plates at 37°C in a humidified 5% CO_2_ air atmosphere in 1 mL of DMEM/F-12 with 10% fetal bovine serum (FBS) (Gibco, Paisley, UK), penicillin (100 U/mL; Gibco, Grand Island, NY, USA), streptomycin (100 U/mL; Gibco, Grand Island, NY, USA) and Fungizone (0.25 µg/ml; Gibco, Grand Island, NY, USA). The biopsies were stimulated with or without modified CpG-ODN for 4 and 24 hours, respectively using 0.1 µM; 0.3 µM and 1.0 µM of CpG-ODN.

### Nasal administrated CpG

Eight patients with CRSwNP were intranasally challenged with either CpG-ODN (*n* = 4) or placebo (*n* = 4). Sterile physiological saline solution (100 µl) containing 50 µM CpG or sterile saline solution (100 µl, placebo) was applied by intranasal spray to both nostrils after exsufflation. Cytosine-phosphate-guanosine-oligodeoxynucleotides (CpG-ODN 2006) were synthesized by DNA Technology (Aarhus, Denmark). The following oligonucleotide sequence was used: 5′ tcgtcgttttgtcgttttgtcgtt 3′. Biopsies from polyp tissue and the turbinate tissue were taken during FESS, 24 hours after the CpG/placebo administration.

### Flow cytometry analysis

The biopsies was placed through a 100 µm cell strainer (BD Falcon), into DMEM/F-12 (1X) containing 10% FBS and incubated at RT for 5 min. The cells were washed and centrifuged after which the supernatant was aspirated and discarded.

The Vybrant Apoptosis Assay Kit #3 from Molecular Probes (Eugene, OR) was used to assess the percentages of viable cells (>80%). The epithelial cells, 20 000–30 000/sample, were subsequently gated based on their EpCAM expression. The cells derived from the biopsies were ∼50% epithelial cells. These cells were analysed for further receptor expression on LRSFortessa analyser (BD, San Jose, USA). The following mouse monoclonal antibodies were used for staining: TLR3-PE (clone 40C1285.6), TLR7-AF488 (clone IMG4G6), TLR8-FITC (clone 44C143), TLR9-AF647 (clone 26C593.2) (Imgenex, San Diego, CA, USA), EpCAM-PerCP-Cy5.5 (clone EBA-1) (Beckman Coulter, Marseille, France), unlabeled VEGFR2 (clone KDR/EIC) (Abcam, Cambridge, UK) were used for staining together with phycoerythrin (R-PE) mouse IgG1 labeling kit (Molecular Probes). Isotype controls relevant for each antibody were used for background staining. IntraPrep Permeabilization Reagent kit (Immunotech, Beckman Coulter, Marseille, France) was used to detect the intracellular TLRs, according to instructions of the manufacturer. For both extracellular and intracellular staining, cells were incubated with antibodies or appropriate isotype controls for 20 min at room temperature, thereafter washed and resuspended in phosphate buffered saline (PBS) (Gibco, Paisley, UK). Data were analysed with FlowJo Analysis Software (©Tree Star, Inc, Ashland, USA).

### Multiplex cytokine measurement

Cytokines in the supernatants from the *in vitro* stimulations were measured using the Human Cytokine Standard 17-plex (Bio-Rad Laboratories, Inc, Corp., Hercules, USA) and quantified on the Luminex200 system. The analysis was carried out according to manufacturer's instructions. Briefly, specific antibodies labeled with spectrally encoded beads were applied to the samples and standards. After incubation, the beads were washed and mixed with specific biotinylated detector antibodies. Subsequently, excess of biotinylated antibodies was removed by a washing step, followed by addition of Streptavidin-R-phycoerythrin (R-PE), in order to conjugate and label the detector antibodies. By monitoring the spectral properties of the beads and the amount of associated R-PE fluorescence by the Bio-Plex System (Bio-Rad Laboratories, Hercules, USA), the concentration of the cytokines could be determined. The cytokines measured were: granulocyte-colony stimulating factor (G-CSF; 1.7 pg/ml), granulocyte monocyte-colony stimulating factor (GM-CSF; 2.2 pg/ml), interferon-gamma (IFN-γ; 6.4 pg/ml), IL-1β (0.6 pg/ml), IL-2 (1.6 pg/ml), IL-4 (0.7 pg/ml), IL-5 (0.6 pg/ml), IL-6 (2.6 pg/ml), IL-7 (1.1 pg/ml), IL-8 (1.0 pg/ml), IL-10 (0.3 pg/ml), IL-12p70 (3.5 pg/ml), IL-13 (0.7 pg/ml), IL-17 (3.3 pg/ml), monocyte chemotactic protein-1 (MCP-1: CCL2; 1.1 pg/ml), macrophage inflammatory protein 1-β (MIP-1β: CCL4; 2.4 pg/ml) and tumor necrosis factor-α (TNF-α; 6.0 pg/ml).

### Statistical analysis

Statistical analysis was performed using Graphpad Prism 5.01 (San Diego, Calif). Mean is represented by a horizontal line for data presented as individual values. Remaining data is represented as mean ± SEM. *N* equals the number of independent donors. For parametric data, statistical differences were determined using unpaired *t*-test (for two sets of data) or one-way repeated measures analysis of variance together with Dunnett's post test (for more than two sets of paired data). Non-parametric data were analysed with a Kruskal-Wallis test together with a Dunn's multiple comparisons post test (for more than two sets of paired data). A *P* value of 0.05 or less was considered statistically significant.

## Results

### Mucosal TLR expression

**Figure 1:**
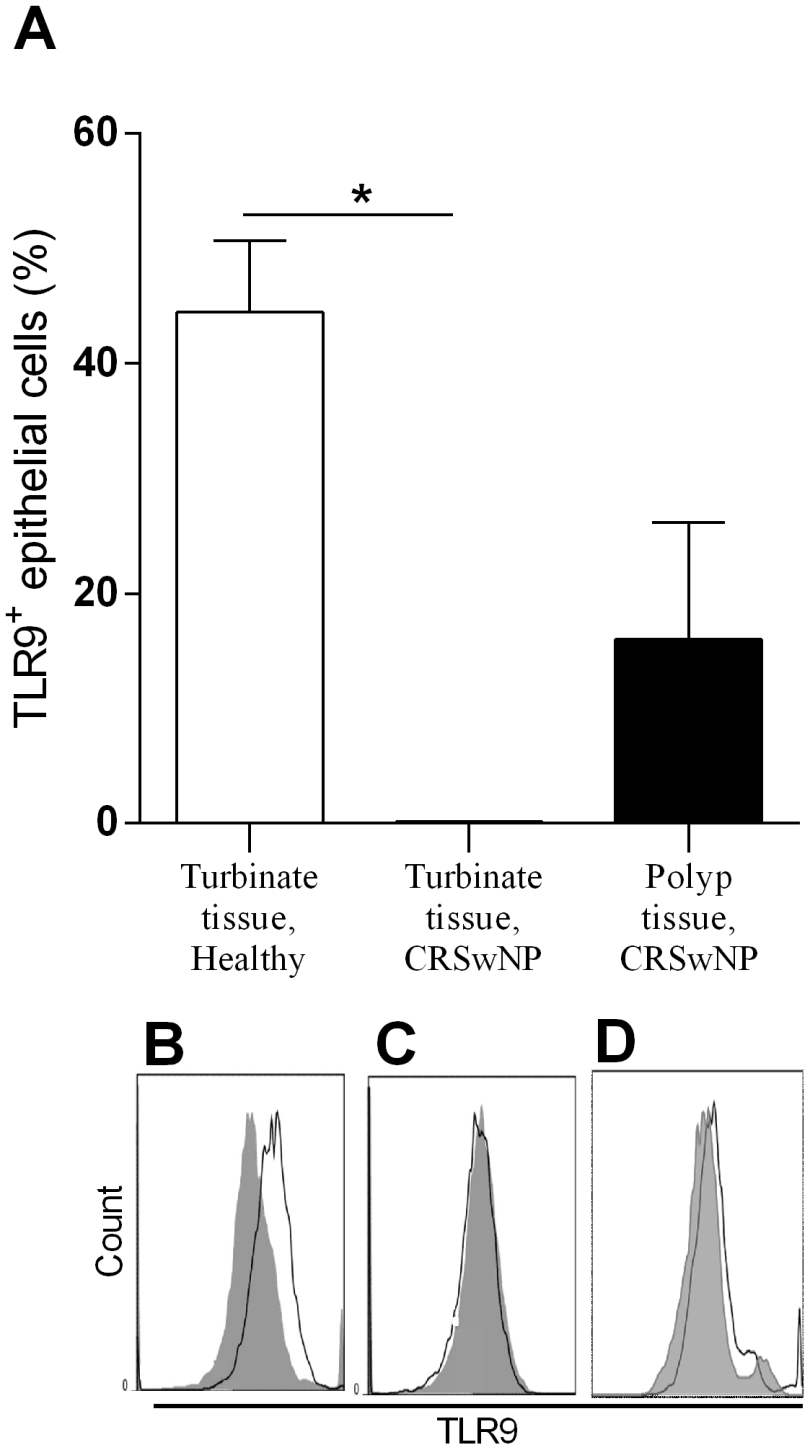
Epithelial TLR9 expression in turbinate tissue from healthy controls as well as turbinate tissue and polyp tissue from patients with CRSwNP. Expression of TLR9 on turbinate epithelial cells from healthy controls compared to turbinate and polyp epithelial cells from patients with CRSwNP, *n* = 5 (**A**). Intracellular staining for TLR9 (open histogram, black line) and isotype control (filled histogram) on turbinate epithelial cells from a healthy control (**B**), turbinate epithelial cells (**C**) and polyp epithelial cells from a patient (**D**), analysed using flow cytometry. Results are presented as mean ± SEM, **P<0.01.

The first set of experiments compared the basal expression of virus-recognizing TLRs in turbinate epithelial cells from patients with CRSwNP, with the TLR expression found in turbinate epithelial cells from healthy controls. TLR3, TLR7 and TLR8 were seen in equal amounts in both groups (data not shown). In contrast, no TLR9-expressing epithelial cells were detected in the turbinate tissue from patients, compared to the significantly higher percentage of turbinate epithelial cells expressing TLR9 from healthy controls (Figure 1A). The polyp tissue demonstrated an increased percentage of epithelial cells expressing TLR9 compared to turbinate tissue from the corresponding patients, although this did not reach significance (Figure 1A–D).

### Effects of CpG stimulation on mucosal TLR9 expression

**Figure 2:**
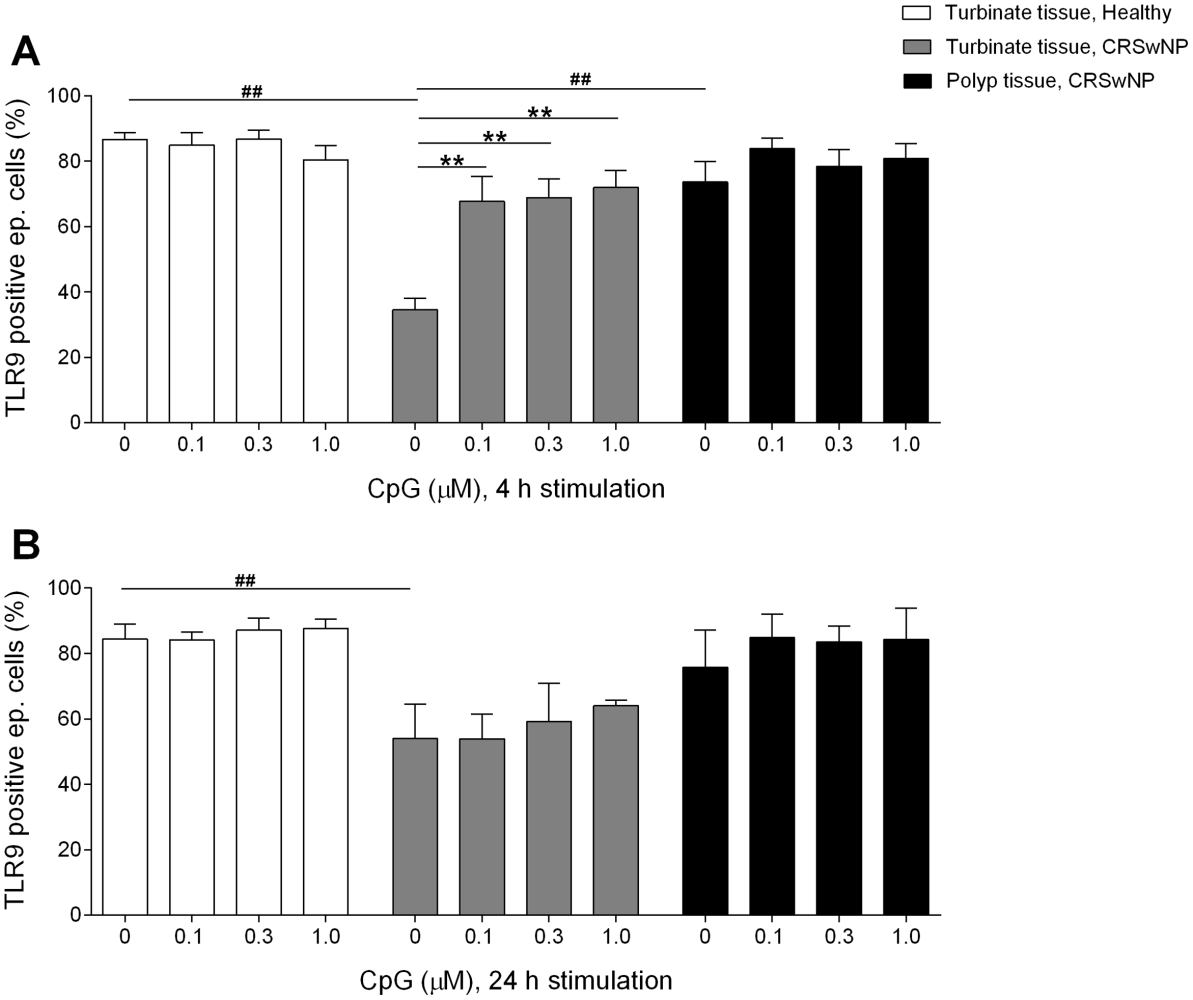
Epithelial TLR9 expression after CpG stimulation. Epithelial TLR9 expression after 4 hours (**A**) and 24 hours (**B**) of culture with vehicle/CpG (0.1 µM; 0.3 µM and 1.0 µM). Expression on turbinate epithelial cells from healthy controls, turbinate and polyp epithelial cells from patients with CRSwNP, analysed using flow cytometry. Results are presented as mean ± SEM, *n* = 6, **P<0.01 (unstimulated vs. CpG stimulated), ##*P*<0.01 (unstimulated turbinate tissue from patients vs. unstimulated turbinate tissue from healthy controls and polyp tissue from patients).

To investigate if CpG impacts the TLR9 expression on epithelial cells, turbinate tissue from healthy controls and turbinate tissue and polyp tissue from patients with CRSwNP were cultured with increasing concentrations of CpG during 4 or 24 hours. Initially, epithelial cells from the turbinate tissue from patients with CRSwNP showed a significantly lower percentage of cells expressing TLR9 compared to turbinate tissue from healthy controls and polyp tissue from patients with CRSwNP (Figure 2A). The percentage of epithelial cells from the turbinate tissue from patients with CRSwNP expressing TLR9 significantly increased as a result of the 4 hours CpG stimulation. Incubation with CpG did not affect the percentage of epithelial cells expressing TLR9 in the turbinate tissue from healthy controls nor did it affect percentage of epithelial cells expressing TLR9 in the polyp tissue itself (Figure 2A). After 24 hours, epithelial cells from the turbinate tissue from patients with CRSwNP still showed a significantly lower percentage of cells expressing TLR9 compared to turbinate tissue from healthy controls (Figure 2B). Incubation with CpG for 24 hours did not significantly affect the TLR9 expression in any of the tissues (Figure 2B).

### Effects of CpG on cytokine release

To analyse the cytokine/chemokine release as well as the effects of CpG stimulation on cytokine/chemokine release in turbinate tissue from healthy controls and turbinate tissue as well as polyp tissue from patients with CRSwNP, supernatants from all experiments were studied. The release of IL-5 in polyp tissue from patients with CRSwNP (6.9±1.2 pg/ml) were significantly higher compared to the release in turbinate tissue from both healthy controls (0.6±0.0 pg/ml, P<0.001) and patients with CRSwNP (0.7±0.1 pg/ml, *P*<0.001). A similar cytokine pattern was seen with IL-10, where the release of IL-10 in polyp tissue from patients with CRSwNP (70.8±9.6 pg/ml) were significantly higher compared to the release in turbinate tissue from both healthy controls (2.3±0.4 pg/ml, *P*<0.001) and patients with CRSwNP (6.9±3.7 pg/ml, *P*<0.001).

**Figure 3:**
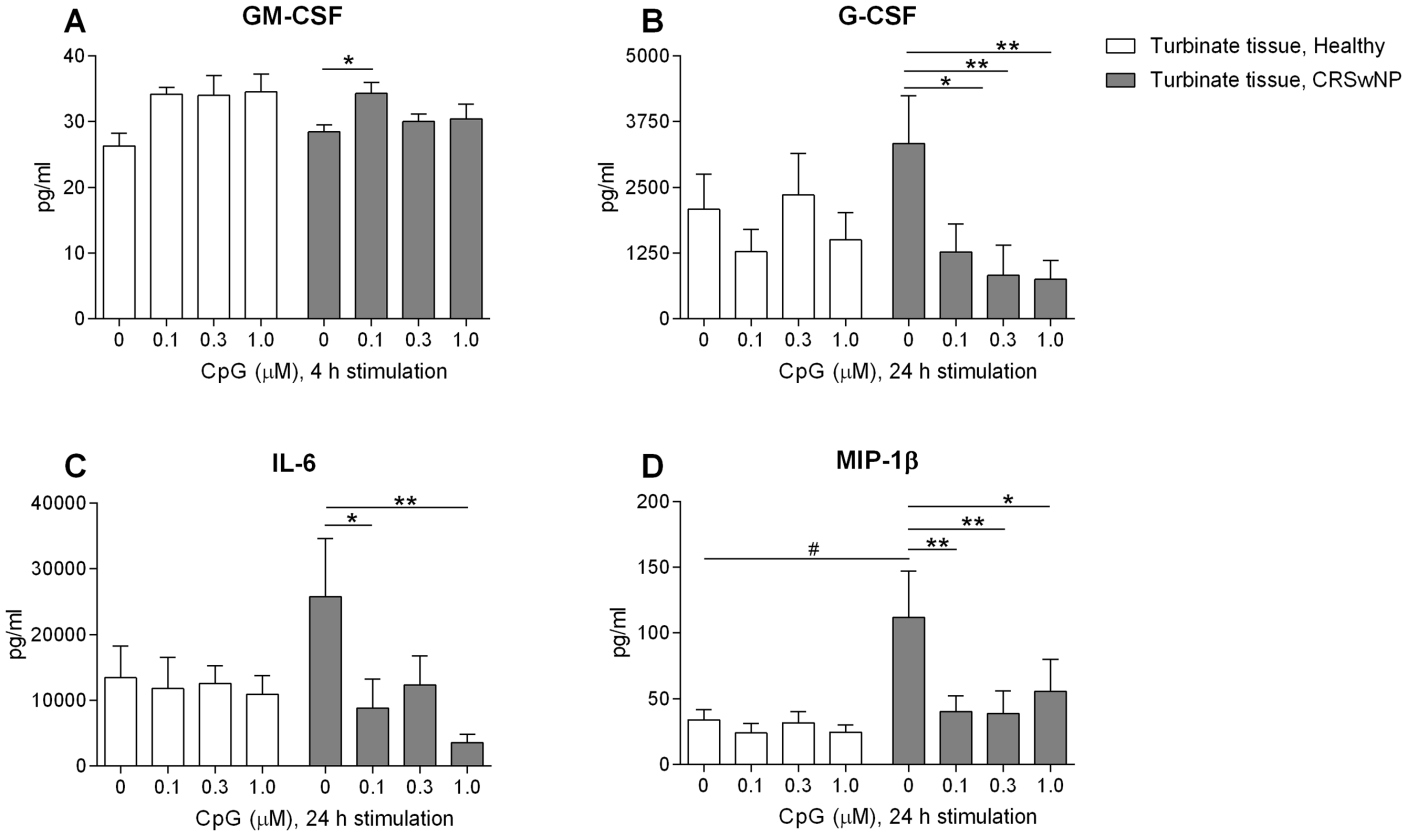
Cytokine secretion from the nasal mucosa after CpG stimulation. CpG induced/reduced cytokine release in culture with turbinate tissue from healthy controls and turbinate tissue from patients with CRSwNP. Samples analysed after culture of 4 hours (*n* = 4) or 24 hours (*n* = 5–8). Levels of GM-CSF (**A**), IL-6 (**B**), G-CSF (**C**) and MIP-1β (**D**) were analysed with Luminex. Data presented as mean ± SEM, *P<0.05; **P<0.01 (unstimulated vs. CpG stimulated), #*P*<0.05 (unstimulated turbinate tissue from healthy controls vs. unstimulated turbinate tissue from patients).

Further, 4 hours of CpG stimulation gave a small increase in GM-CSF release although this effect was not concentration-dependent and was only significant at the lowest dose (Figure 3A). No significant increase in GM-CSF release was seen in healthy turbinate tissue (Figure 3A). In the polyp tissue, no significant GM-CSF alteration was seen after CpG stimulations (0 µM, 81.2±14.8 pg/ml; 0.1 µM, 82.4±25.2 pg/ml; 0.3 µM, 88.8±16.3 pg/ml; 1.0 µM, 70.6±24.8 pg/ml). The following cytokines and chemokine analysed were unaffected by CpG stimulation for 4 hours (all tissues), G-CSF, IFN-γ, IL-1β, IL-2, IL-4, IL-5, IL-6, IL-7, IL-8, IL-10, IL-12, IL-13, IL-17, MCP-1, MIP-1β and TNF-α (data not shown).

After 24 hours of incubation the levels of IL-6, G-CSF and MIP-1β all tended to be higher under control condition in the turbinate tissue from patients as compared to the levels demonstrated in turbinate tissue from healthy controls (Figure 3B–D). CpG stimulation for 24 hours seemed to reduce them all towards the levels demonstrated in healthy controls (Figure 3B–D). Incubation with CpG during culture of turbinate tissue from healthy controls did not affect the release of IL-6, MIP-1β and G-CSF (Figure 3B–D). In the polyp tissue, no significant alteration was seen after CpG stimulations analysing IL-6 (0 µM, 22598±10713 pg/ml; 0.1 µM, 19157±5332 pg/ml; 0.3 µM, 23592±5769 pg/ml; 1.0 µM, 27600±9155 pg/ml), MIP-1β (0 µM, 392±93 pg/ml; 0.1 µM, 343±31 pg/ml; 0.3 µM, 409±119 pg/ml; 1.0 µM, 264±95 pg/ml) or G-CSF (0 µM, 7404±2409 pg/ml; 0.1 µM, 4652±1039 pg/ml; 0.3 µM, 7388±1172 pg/ml; 1.0 µM, 7933±3819 pg/ml). The following cytokines and chemokine analysed were unaffected by CpG stimulation for 24 hours (all tissues), GM-CSF, IFN-γ, IL-1β, IL-2, IL-4, IL-5, IL-7, IL-8, IL-10, IL-12, IL-13, IL-17, MCP-1 and TNF-α (data not shown).

### Effects of CpG stimulation on VEGFR2 expression

**Figure 4:**
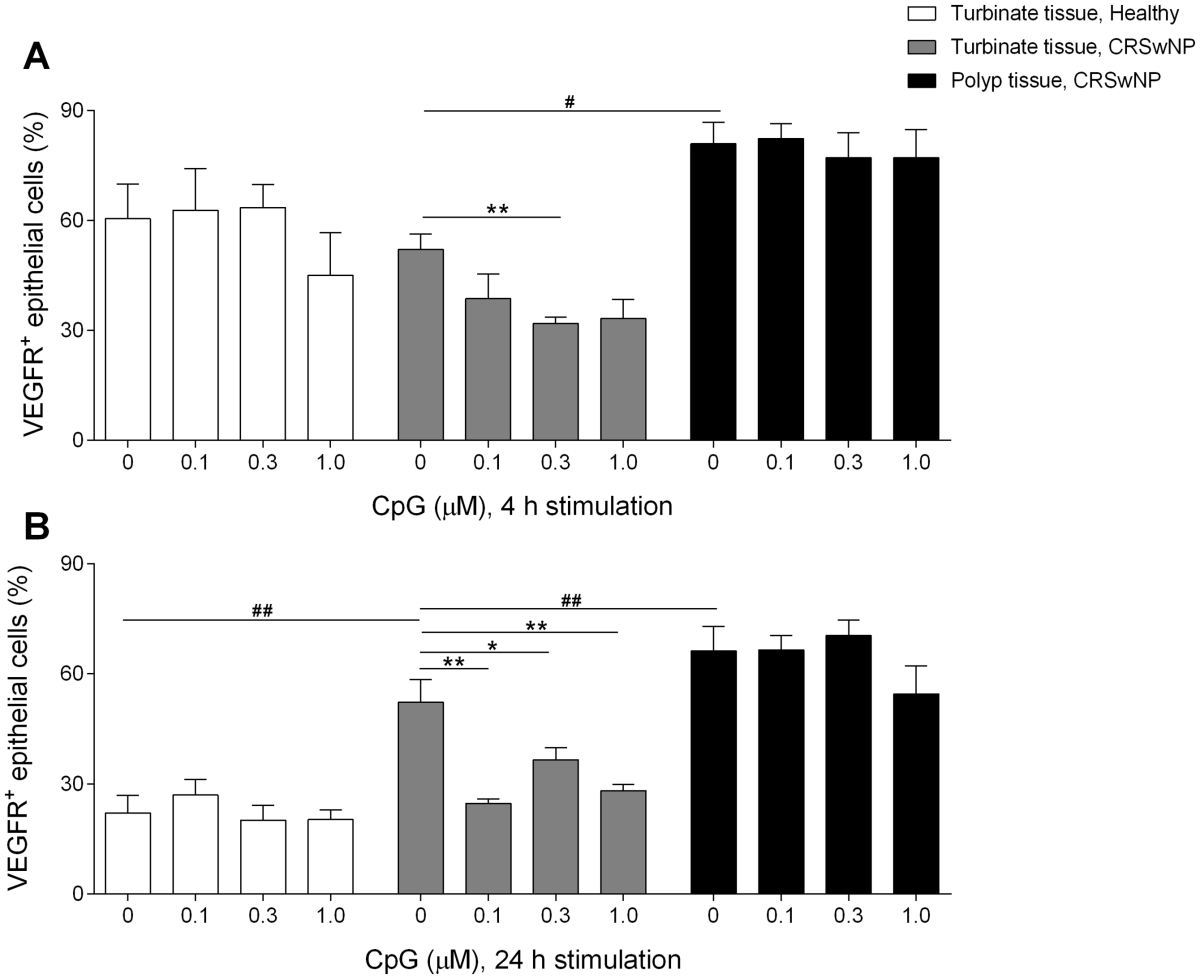
Percentage of cells that express VEGFR2 after CpG culture. Epithelial VEGFR2 expression after 4 hours (**A**) and 24 hours (**B**) of culture with vehicle/CpG (0.1 µM; 0.3 µM and 1.0 µM). Expression on turbinate epithelial cells from healthy controls, turbinate and polyp epithelial cells from patients with CRSwNP, analysed using flow cytometry. Results are presented as mean ± SEM, *n* = 5, *P<0.05, **P<0.01 (unstimulated vs. CpG stimulated), ##*P*<0.01 (unstimulated turbinate tissue from patients vs. unstimulated turbinate tissue from healthy controls and polyp tissue from patients).

To explore whether CpG affected polyp development, vascular endothelial growth factor receptor 2 (VEGFR2) expression was studied on the epithelial cells. Both 4 and 24 hours CpG culture of the turbinate tissue from patients with CRSwNP resulted in reduction in the percentage of epithelial cells that express VEGFR2. In contrast, the VEGFR2 expression in turbinate epithelial cells from healthy controls as well as in epithelial cells from polyp epithelial cells remained unaffected by CpG stimulation (Figure 4A–B).

### Effects of nasal challenge with CpG

**Figure 5:**
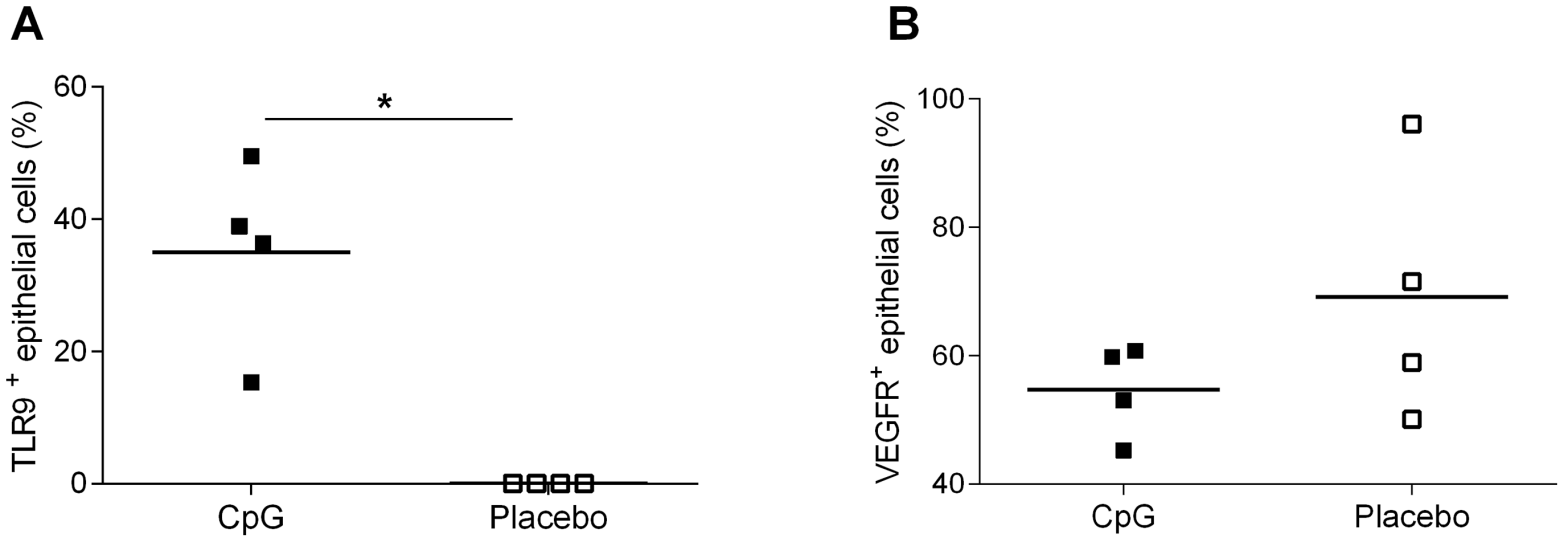
TLR9 and VEGFR2 expression after nasal challenge with CpG. Eight patients with CRSwNP were nasally challenged with either CpG (*n* = 4) or placebo (*n* = 4). Biopsies from the turbinate tissue were obtained during FESS, 24 hours later. The nasal turbinate epithelial cells were stained intracellularly for expression of TLR9 (**A**) and VEGFR2 (**B**) and analysed using flow cytometry. Results are presented as mean ± SEM, *P<0.05.

Eight patients with CRSwNP in total were intranasally challenged with CpG or placebo 24 hours prior to FESS. We could demonstrate a high percentage of TLR9 expressing epithelial cells in the turbinate tissue in the active CpG-treated group. In the placebo group, almost none of the corresponding epithelial cells expressed TLR9 (Figure 5A). Similar to the *in vitro* experiments presented above, the active CpG treatment appeared to some extent decrease the number of VEGFR2 positive epithelial cells, this by 21 %, 24 hours after active CpG treatment, although this did not reached statistical significance (*P* = 0.242) (Figure 5B). No significant reduction was seen in the polyp tissue (data not shown).

## Discussion

The present paper reveals several important differences between the mucosa bordering the polyp and the polyp tissue itself. Of special interest is that TLR9 expression was detectable in CRS polyps and in the nasal mucosal from healthy volunteers, but the expression was almost absent in epithelial cells from the mucosa close to the anatomical origin of the polyps. However, TLR9 expression could be reconstituted in the deficient mucosal epithelium bordering the polyp following CpG stimulation, both *in vitro* and *in vivo*. Stimulation also decreased the expression of VEGFR2 on the epithelial cells in turbinate tissue from patients with CRSwNP. Four virus-recognizing TLRs were measured; TLR3, TLR7, TLR8 and TLR9, but only TLR9 was found to be absent in the mucosa bordering the polyp. This highlights the importance of TLR9 expression in the airway epithelium from patients with CRSwNP.

TLR9 activation induces a Th1-biased immune response that is known to suppress Th2-related activities associated with CRSwNP. This response is initiated upon recognition of CpG in the endosome after a non-specific uptake into the cell [20]. Several studies have demonstrated immunostimulatory properties of CpG *in vitro* in humans [21], [22] as well as *in vivo* in mice [23], [24]. These findings demonstrate the ability of CpG to strongly activate different cell types to promote a Th1-biased immune response [13], [22]. An almost absent expression of TLR9 was seen in epithelial cells from the mucosa bordering the polyp, but stimulation with CpG, both *in vitro* and *in vivo,* upregulated this expression. The discrepancies in TLR9 expression between freshly analysed biopsies and *in vitro* unstimulated biopsies is probably related to serum content in the media of the latter. However, a similar TLR9 upregulation could not be induced in polyp derived epithelial cells. The upregulation of TLR9 in epithelial cells from the turbinate tissue bordering the polyp may have been due to the low, but probably present, TLR9 expression responding to CpG-ODN. However, the mechanism by which CpG-ODN may upregulate TLR9 on epithelial cells needs to be further evaluated.

It is clinically well-established that viral infections aggravate inflammation causing concomitant polyp growth [25]. TLR9 is known to respond to such microbial intrusion by initiating a host response reaction [20]. The lack of epithelial TLR9 activity at the site of polyp growth might therefore contribute to the virus induced polyp development seen among patients with CRSwNP. The restored and activated TLR9 receptor, as the result of the CpG-ODN stimulation, may improve the immune defense of the turbinate tissue that could lead to viral infection in smaller extent causing polyp growth. Studies in mice have demonstrated that the innate immune defenses activated by CpG-ODN, given by injection, inhalation, or even by oral administration, can protect against a wide range of viral, bacterial, and even some parasitic pathogens, such as *Listeria monocytogenes*, *Mycobacterium tuberculosis*, herpes simplex virus and respiratory syncytial virus [26]. The CpG-ODN treatment could, through the activated TLR9, provide temporary protection against diverse pathogens. However, interaction with other functions of the adaptive immunity resulting in polyp growth inhibition cannot be ruled out. The small upregulation of TLR9 expression due to serum in the media seen in all the tissues were of the same magnitude in all tissues specimens tested and it did not affect the CpG upregulated TLR9 expression. The increased percentage of cells that express TLR9 in the turbinate tissue from patients with CRSwNP was also confirmed *in vivo* after 24 hours. Kodama et al. have demonstrated that weekly administrated CpG in the nasal cavity appears unharmful to mice [24]. Equivalently, we have previously registered no apparent side effects in conjunction with nasal CpG challenges of healthy volunteers [27]. Our data along with previous studies may impact the idea of CpG derived ligands as a future therapeutic drug for patients with CRSwNP.

The nasal mucosa from patients with CRSwNP in the western world is characterized by eosinophilic predominance and a Th2 cytokine profile [28]. The present study demonstrates different cytokine profiles, under control conditions, in the polyp tissue compared to both the turbinate tissue from patients with CRSwNP and from healthy control. IL-5 is an important cytokine in patients with CRSwNP as it is responsible for delaying eosinophilic apoptosis in nasal polyps [29]. The anti-inflammatory nature of IL-10 also contributes to polyp formation by enhancing local Th2 inflammation and the associated tissue damage [30]. Both cytokines were higher in polyp tissue compared to the turbinate tissue from patients with CRSwNP. Other groups have demonstrated a higher release of IL-5 and IL-10 in polyp tissue than in turbinate tissue from healthy controls [30], [31]. The development of an acute sinusitis is usually preceded and accompanied by inflammation and cytokine secretion from the nasal mucosa [18]. Dysregulation of the innate antimicrobial processes that normally protect the host can promote microbial colonization or infection [32]. Similarly, failure to limit the virus induced innate immune responses can create a persistent damaging inflammatory process. In the absence of TLR9 activity and a robust Th1 inflammatory activation, an eosinophilic Th2-skewed inflammation can arise, in our study demonstrated by upregulated IL-5, IL-6, IL-10, G-CSF and MIP-1β. This impaired microbial defense could allow the microorganisms to become permanently deposited in the nasal mucosa [32].

The Th2 cytokine profile is also known to be suppressed by TLR9 induced Th1 activity [11]. Stimulation of turbinate tissue from patients with CRSwNP with CpG during 4 hours resulted in a small increase in GM-CSF release. In line with this, GM-CSF has been demonstrated to enhance neutrophilic responses to CpG through a TLR9-dependent mechanism [33]. After 24 hours of CpG stimulation, the high levels of IL-6, G-CSF and MIP-1β of the turbinate tissue from patients with CRSwNP had decreased, reaching levels corresponding to the release from turbinate tissue from healthy volunteers. In line with this, Zhang et al. have demonstrated an increased IL-6 release in polyp tissue from patients with CRSwNP [34]. It is possible that increased levels of IL-6, seen in turbinate tissue from patients with CRSwNP, may be responsible for the recruitment and retention of T cells seen among patients with CRSwNP [35], [36]. As the IL-6 levels decreased after CpG stimulation, this could be an efficient way of preventing this recruitment. Though TLR9 primarily is expressed on B cells and plasmacytoid dendritic cells (pDCs), it has also been detected on epithelial cells [37], [38]. As the B cell numbers are comparably low in the nasal mucosa we propose that epithelial cells that are high in numbers, have an important role in the cytokine secretion [19], [39]. Hence, the reported cytokine secretion could be emanating from any of these cells in the nasal mucosa. Administration of a CpG-ODN activates pDCs to promote Th1 adaptive immune responses. This is reflected in B cells and pDCs showing increased expression of costimulatory molecules, upregulation of the chemokine receptor, CCR7, and secretion of Th1-promoting chemokines as well as cytokines, such as MIP-1β and IFN-γ–inducible protein-10 [40]. Although no mouse model currently exists for CRSwNP, mice with upper and lower allergic Th2 inflammation exhibit a decreased expression of innate immune genes and reduced capacity to clear infection [41], [42]. It is important to recognize that, in contrast to what was seen in epithelial cells from turbinate tissue, no cytokine reduction was found in the polyp tissue as a result of the CpG stimulation. The downregulation of cytokine release to the level seen in healthy controls could further support the use of CpG for immunotherapy against infectious disease.

The present finding of high VEGFR2 expression on epithelial cells in turbinate tissue from patients with CRSwNP compared to the low expression found in turbinate tissue from healthy controls, after 24 h of culture, suggests that VEGFR2 is important in polyp development. Nasal polyp growth requires epithelial proliferation and accumulation of extracellular matrix [1], [43], [44]. For optimal function these growth processes are in need of an increased vascular supply, something that could be regulated by VEGFR located on epithelial cells [45]. VEGFR participates in angiogenesis by enhancing proliferation, migration and vascular permeability [46]. The VEGFR also regulates capillary and basal membrane permeability in nasal polyps and increased VEGFR expression can cause edema and polyp growth [47]. Soluble VEGF and VEGFR2 have been shown to be upregulated on epithelial cells in nasal polyp tissue [48], [49]. Bobic et al. show that VEGFR2 not is upregulated in nasal polyps compared to turbinate tissue from controls, but these polyps originate from patients that had taken nasal corticosteroids [50]. The patients in our study did not take steroids for 6 (topically) and 12 weeks (systemically) prior to FESS. This indicates that VEGFR2, compared to VEGFR1, is affected by steroid treatment and could thereby be an important player in polyp growth.

Further, CpG stimulation resulted in a clear downregulation of VEGFR2 on epithelial cells in turbinate tissue from patients with CRSwNP after both 4 and 24 h in vitro. Locally administrated CpG reduced the VEGFR2 expression on turbinate epithelial cells from patients with CRSwNP by 21 %, 24 hours after active CpG treatment. No such reduction was seen in the polyp tissue. Viral infections have been demonstrated to further upregulate the VEGFR expression in epithelial cells [51]. The finding of high VEGFR2 expression on epithelial cells from turbinate tissue and a downregulation following CpG stimulation further demonstrates the function of CpG as a restrictor of polyp growth. Reduced VEGFR expression decreases microvascular permeability, leading to less plasma proteins accumulation in the extracellular matrix, thereby contributing to polyp growth inhibition [47]. The VEGFR2 expression in healthy controls was unaffected by CpG stimulation and the generally high level found in the turbinate tissue from patients with CRSwNP further indicates the importance of an inhibitory role of CpG in polyp growth.

To summarize, this paper investigates epithelial cells derived from an area close to the anatomical origin of the polyps in patients with CRS. A deprived expression of TLR9 was detected in this area. This deficiency was seen neither in the polyp tissue nor in the healthy turbinate tissue. CpG stimulation upregulated the TLR9 expression and downregulated the VEGFR expression, suggesting a correlation between TLR9 and VEGFR. It is therefore tempting to assume a role for TLR9 activation in restricting polyp growth. The demonstrated ability of CpG to upregulate and activate TLR9 has to be considered important in the struggle to reduce or inhibit return of polyps after surgery. However, even more important for our future CRS research is the finding that the polyp itself does not necessarily reflect the inflammatory conditions at its nasal epithelial growth zone. Hence, some of our previous conclusions based on polyp findings might have to be supplemented and revised.
